# William Wimsatt, Donald Campbell, and the Idea of Robustness

**DOI:** 10.1007/s10441-026-09543-6

**Published:** 2026-10-04

**Authors:** Morgan Thompson

**Affiliations:** Department of Philosophy, Northwestern University, Evanston, USA

**Keywords:** Robustness, Triangulation, William Wimsatt, Donald Campbell, Construct validity

## Abstract

In this paper, I chart the influence of social scientist Donald Campbell’s work on William Wimsatt’s ideas of robustness reasoning. One reason is that Campbell’s work is less familiar in the broader philosophy of science literature. Another is that Wimsatt’s famous 1981 paper on robustness was originally published in a Festschrift for Campbell and cited the three major works discussed here: on pattern-matching, on definitional operationism, and on the multitrait, multimethod matrix analysis of construct validity. I highlight some questions in contemporary debates in robustness reasoning: what is (are) the function(s) of robustness, the problem of discordant evidence, and the unification of robustness reasoning. I argue that some debates can be traced back to ideas present in both Campbell’s and Wimsatt’s work. Here I appeal to some unpublished notes and correspondence from Campbell as support and to coherence with Wimsatt’s larger philosophical framework. I propose that some resolution can be found there as well, through Wimsatt’s idea of robustness as a heuristic.

## Introduction

William Wimsatt’s work stretches across disciplinary boundaries and defies tidy categorization. Much of his work concerns topics central in philosophy of biology: how ought one think about levels of reality? What are successful reasoning strategies employed by biologists? How do reductive approaches constrain or enable our understanding of complex systems? His answers to these questions draw inspiration from engineering, early computer science, and psychology. In general, Wimsatt’s work approaches problems that scientists encounter (even if not described in a philosopher’s jargon) and that a humanistic philosophy of science can help address.

Wimsatt completed a bachelor’s at Cornell University in philosophy 1965. He finished a PhD at the University of Pittsburgh in 1971. For his dissertation, Wimsatt asked biologist Richard Lewontin to be an outside committee member, not a philosopher of biology (Wimsatt 1982). This choice foreshadows a career long effort to engage deeply with the biological sciences and with scientists. Wimsatt accepted a postdoctoral position at the University of Chicago working with Richard Lewontin. There he met many scientists who influenced his work, including Donald Campbell (by then at Northwestern) and Richard Levins (Wimsatt 1982). After this postdoctoral position, Wimsatt became a faculty member at Chicago and spent his career there with appointments in Philosophy, Biology, and on the Committee on Conceptual Foundations of Science. Teaching in the Biology department afforded Wimsatt the time and commitment to stay abreast of cutting-edge debates in biology, embodying practices now explicitly cultivated among philosophers of science in practice (Wimsatt 1982).

One of Wimsatt’s early supporters during his career was the prolific social scientist Donald T. Campbell. Campbell’s work touched on methodology in psychology, evolutionary epistemology, the structure of science, and social attitudes research. Although Campbell never considered himself a philosopher, he was deeply embedded in philosophy of science’s intellectual and social networks (Heyes 2001). Wimsatt organized a symposium at the first Society for Philosophy and Psychology (SPP) meeting in 1981 that featured Campbell, Quine, and Sellars. Both Campbell and Wimsatt held positions at Chicago, though in different departments and at different times. They met in 1971 when Campbell gave a lecture on George Williams in Ralph Burhoe’s seminar on science and religion at Chicago. Wimsatt had just reviewed Williams’ book for *Philosophy of Science* (Wimsatt 1970). Though Campbell left for Chicago for Northwestern in 1953 (Campbell 1981/1988), they interacted frequently. Both thinkers contributed to evolutionary epistemology, a naturalistic approach to epistemology on which (i) epistemology is continuous with the sciences and (ii) the processes of natural selection also act to select among potential learning strategies without guaranteeing success in generating knowledge in advance. Throughout Wimsatt’s career, Campbell wrote letters in support of various job applications, promotions, or opportunities (Campbell n.d.-d). Campbell clearly thought highly of Wimsatt’s work and held him as one of the best evolutionary epistemologists (or “natural epistemologists”) during his career; in fact, Campbell wrote in two different letters of support from 1981 that he held Wimsatt in better regard than Quine as an evolutionary epistemologist (Campbell, n.d.-d). Meanwhile, Wimsatt (along with Donald Fiske) nominated Campbell for an honorary degree from Chicago in 1981. Campbell received the honorary degree despite having moved to Northwestern at the time due to concerns about receiving tenure at Chicago due to extraordinary pressures in the Psychology department at the time (Campbell 1981/1988).

At Northwestern, Campbell co-taught a course with the Philosophy Department called “Knowledge Processes” from 1963 to 1971. His notes from “Knowledge Processes” and in preparation of his research papers, he engaged with the works of Plato, Kant, Hume, Frege, Hempel, Hanson, Neurath, Kuhn, Lakatos, Feyerabend, and Polyani (Campbell, n.d.-d). In later iterations of the course, Wimsatt came up from Chicago for private conversations and to guest lecture (Campbell 1988a, b, 22). This seminar was notable as a version of “naturalistic epistemology” rather than fitting into the logical positivist framework that was dominant across Northwestern departments at the time. In notes from the course, students were learning about Kant, Hume, Popper, theory-laden observation, and perception (Campbell, n.d.-d). No doubt Wimsatt fit well into such an interdisciplinary course that emphasized fallibilist epistemology.

Wimsatt too notes their intellectual relationship. In fact, Wimsatt takes it to be one of his achievements having convinced Herbert Simon and Donald Campbell that they had “consilient evolutionary visions” of the sciences that respect both humanistic and Darwinian perspectives (2007, ix). Of this, Wimsatt and Campbell again share common skill and intentions to bring an interdisciplinary movement together by building intellectual alliances between others. To Wimsatt’s interpretation, this may have resulted in Campbell’s 1974 paper giving Popper too much credit for certain ideas. Campbell was also a humble builder of intellectual community; Wimsatt recalls asking for a print copy of one’ of Campbell’s papers, to which he received Campbell’s paper and papers by three other scholars.^1^ Among other influential figures, Wimsatt mentions “[d]eeply informative influences of Herbert Simon, Donald Campbell, Richard Lewontin, and Richard Levins, and Frank Rosenblatt” (the grandfather of modern AI), relationships which all began in the mid-1960s (2007, ix). While both Campbell and Wimsatt were broadly “evolutionary epistemologists” (since expanded in Wimsatt’s work on cultural evolution, e.g., Love and Wimsatt 2019), their work has another line of connection in robustness reasoning. Wimsatt notes that his 1981 paper on robustness began from “seeds planted around 1970 by the writings and ideas of Donald Campbell and Richard Levins” (2007, 368). In fact, Wimsatt’s famous 1981 paper was published in Donald Campbell’s Festschrift.

Continuing this tradition, I offer here a Festschrift contribution about a Festschrift paper. In this paper, I will chart the influence of Campbell’s work on Wimsatt’s ideas of robustness reasoning. One reason is that Campbell’s work is less familiar in the broader philosophy of science (except in small pockets of those working on construct validity or evolutionary epistemology) and in the proceeding literature on robustness analysis and methodological triangulation than Levins’ work (e.g., Levins 1966). I will also highlight some questions in contemporary debates in robustness reasoning: the function(s) of robustness, the problem of discordant evidence, and the unification of robustness reasoning. I argue that some of these debates can be traced back to ideas present in both Campbell’s and Wimsatt’s work. I propose that some resolution can be found there as well, though Wimsatt’s idea of robustness as a heuristic.

## Robustness as a Guide for Epistemically Limited Beings

Robustness is broadly the idea that multiple, diverse but fallible methods can provide more reliable support for a claim than any method alone. It is one way to identify trustworthy and reliable claims about the world, despite various sources of fallibility—our own cognition and our understanding of our methods for investigating the world. One basic way to support the idea of robustness as a successful strategy for identifying reliable claims is that our methods are subject to error—both known and unknown. Individually we can imagine that each method is a single fiber. Many fibers with different orientations can form an interconnected structure (Peirce 1958, 40–41; cited in Thagard 2009).^2^ While any individual fiber may be broken, together they are stronger. Even if one fiber were to break, the others may hold. Similarly, each method may have different biases and assumptions, but if there is sufficient diversity among the methods, then their agreement can be taken to give reliable support to the result.

Wimsatt describes robustness as a heuristic used across different scientific contexts, when similar problems arise in different research projects. Wimsatt notes (1981/2007, 63):

All the variants and uses of robustness have a common theme in the distinguishing of the real from the illusory; the reliable from the unreliable; the objective from the subjective; and, in general, that which is regarded as ontologically and epistemologically trustworthy and valuable from that which is unreliable, ungeneralizable, worthless, and fleeting.

So, on Wimsatt’s view, robustness helps researchers to make ontological and epistemic claims about reliability of their methods, distinguishing illusory claims about the reality of phenomena, and also, by comparative cross-checking, to discover the weakness of one another.

Robustness reasoning is well suited to fallible creatures like us. Error always lurks in the background. So, Wimsatt holds “fallible thinkers should avoid long serial chains of reasoning” (1981/2007, 49). Why is this? Despite the support for any given link in a single chain of reasoning, our justification for the link could always be undermined. And if we are wrong, then the whole chain is broken. For Wimsatt, we must consider the possibility that we are wrong, but this view need not lead to a global skepticism. Instead, he developed an account of successful heuristics that have already been in scientific use but had not been schematized until his 1981 paper.

Wimsatt argued robustness can help reduce systematic error specifically. Random noise can be assumed to cancel out in the way that binocular vision removes minor variations in similar images presented to each eye (Wimsatt 1981/2007, 57; discussing Campbell 1966). However, systematic error stemming from any derivational, measurement, or other procedure might influence our result. Robustness can provide a better heuristic guide to trustworthy inferences than relying on a single method alone. It better supports both generalization and prediction (1981/2007, 57).

According to Wimsatt (1981/2012, 44), robustness reasoning often follows these four steps:

1. Analyzing multiple independent derivation, identification, modeling, or measurement procedures.
2. Searching for a feature that is invariant over the results of the procedure.
3. Identifying the scope of invariance and which conditions produce the feature that accounts for it.
4. Explaining any failures of invariance.^3^

The first step requires that researchers have access to multiple means of determination^4^, including means of derivation, identification, modeling, or measurement. There is already a philosophical commitment to the idea that the different means of detection can be used to investigate the same research question—one that rejects a strict reading of operationism.^5^ These multiple means of determination also need to be accessible to the researcher. Even if we agree it is possible to measure the same phenomenon with different tools, we do not always have multiple means available in practice. Finally, these means of determination should be independent or distinct, e.g., in their theoretical or statistical assumptions (Soler 2012; Coko 2020b).

Step 2 tells researchers to examine some feature that remains invariant, despite the differences between the means of detection. One interpretation might hold that invariance in the value of a predicted outcome is successful identification. Another interpretation is that some feature of the means of determination themselves may be invariant. The latter follows Levins’ idea of ‘robustness theorems.’ Weisberg (2006) and others have emphasized that robustness reasoning strategy involves the search for some assumption or aspect of the models that might account for the invariance. When successful, this can fulfill Step 3 as well. It may also undermine strict interpretations of independence when there is a common assumption (Schupbach 2018). For example, if multiple models of predator-prey relations assume infinite populations, then these models would not be independent.

In Step 3, the invariance itself becomes the explanatory target. Suppose the means of detection were experimental. Under which experimental set-ups will the invariant result continue to hold? The researcher can vary many aspects of their experimental protocol or the measurement instrument itself to identify the conditions under which the invariance holds. In many cases, this intentional variation will identify some conditions under which the invariance breaks. So, if the invariant feature is the value of an outcome variable, then the value will vary under the larger set of experimental conditions.^6^ The researchers’ task is then to explain why multiple means of detection under certain conditions produce an invariant outcome, while under other conditions they do not (Step 4). Wimsatt’s steps for robustness do not presume perfect concordance among the measurement results in practice.

Even in this four-step schema, we can see Wimsatt’s commitment to understanding research strategies like robustness from a fallible, human perspective. As limited, biased creatures engaging in epistemic practices, we will inevitably err. Our production of evidence, our inferential reasoning, are both fallible. But importantly for Wimsatt, they are also potentially self-correcting (Wimsatt 2007, 7). Is it best to view robustness reasoning as a heuristic or an algorithm? Wimsatt’s epistemology holds that limited beings like us are best served by heuristics rather than algorithms. When truth-preserving algorithms are applied to a problem, they guarantee a solution. Inspired by Herbert Simon’s work, Wimsatt notes that heuristics do not guarantee a solution, even when correctly applied (Wimsatt 2007, 76–77). However, heuristics have other benefits for us: they are cost-effective in resource usage, systematically biased, and transform problems into “nonequivalent but intuitively relatable” problems (Wimsatt 2007, 77). Heuristics help us build up knowledge over time about the problems to which they can successfully apply. Further, systematic bias is a *benefit* for limited beings, when we can also learn about and from the error. Inspired by his background in engineering, Wimsatt at times speaks of “differential improvement from the current state” (Wimsatt 2023). That is, heuristics offer improvement in resolving problems from our environmental and historical position. Heuristics are also tested over time and generations, so we start from a situated position rather than from scratch each time. So, heuristics are better suited for limited epistemic creatures because they allow (but do not guarantee) success under the conditions in which we tackle epistemic problems.

Wimsatt holds this four-step schema for robustness reasoning characterizes the commonalities across scientific reasoning concerning sameness across some sort of variation: from multimodal sensory detection to different measurement procedures and from different modeling assumptions to different theoretical descriptions of a phenomenon (Wimsatt 2007, 45). Recent philosophers of science have suggested that there are several types of robustness reasoning that can be distinguished. Calcott (2011, 283–284) distinguishes three types: (i) robust theorems that are supported by multiple derivations, (ii) robust phenomena that reliably occur in different contexts (e.g., such as those due to generative entrenchment), and (iii) robust detections that occur when a claim about a property or entity is detected by multiple, independent lines of evidence. Similarly, Eronen (2015) argues that (i) and (iii) are distinct with respect to their ability to justify realist claims.

## Campbell’s Influence on Wimsatt’s 1981 Robustness Festschrift

In this section, I will explain three of Campbell’s papers that had influence on Wimsatt’s ideas and perhaps vice versa. Each paper is one that Wimsatt cites in his work on robustness.

### Pattern Matching

Wimsatt (1981) specifically cites Campbell’s paper on “pattern matching” and object identification as inspiration for his account of robustness reasoning. Appealing to visual perception and object identification, Campbell considers how we come to know anything beyond our immediate sensations. Consider the problem: Humans must build knowledge of a stable object despite differences in immediate visuals from different angles. In practice, researchers often assume that the difficulty involves forming accurate beliefs about the size, shape, and other properties of the object. Yet, as Campbell notes, such research questions rely on the assumption that there is a single object responsible for all these sensations. In ignoring how humans come to this judgment, “one misses some of the wonder of the achievement” (Campbell 1966, 11). Campbell recognizes that “there is always the alterative of inferring several independent causal sources for the several proximal data, rather than inferring a single common source” (Campbell 1966, 11). How do we determine distal knowledge from proximal information? When does our perceptual system presume that visual images of an apple from above and from the side are in fact proximal visual inputs of the same apple rather than different ones? His answer is that we can use pattern-matching. Different perceptual images, while individually fallible, tend to agree on the boundaries of everyday objects. This strategy is why perceptual images of objects are taken to be “veridical rather than illusory” (Wimsatt 2007, 60).

Campbell thought pattern-matching as a cognitive strategy spans all knowledge systems, “including the achievements of scientific theory” (Campbell 1966, 11–12). Campbell viewed scientific knowledge as resulting from the same general processes as perceptual knowledge (Campbell 1974). Assuming a broadly realist view (as both Wimsatt and Campbell do),^7^ human cognitive activities achieve knowledge of both the objects we perceive and scientific phenomena by combining proximate, dissimilar information built on assumptions that could be false.

### Operationism

Campbell’s work engaged with and reacted to the ideas of logical positivism to which many social scientists at Northwestern were committed during his career. In Campbell’s (1969/1988) essay on operationism, he develops his own idea of robustness with multiple operationism. Campbell rejects ‘definitional operationism’, a version of operationism on which operational descriptions of a term uniquely define it. For Campbell, terms denote entities assumed to be real (Campbell 1969/1988, 32). Researchers make use of this by using operational descriptions as “diagnostic of the presence of absence of the object or process” (Campbell 1969/1988, 32). So, any operational description of the entity must be incomplete, given the possibility of unknown attributes of the entity. There must be other operations by which these unknown attributes could be measured. While he never defends his realist commitments, they motivate him to reject definitional operationism.

Campbell provides two arguments from methodological robustness to multiple operationism. First, he offers a theoretical argument (Campbell 1969/1988, 32). Consider a measurement process of a given attribute. On definitional operationism, a single operation (or measure) uniquely defines a concept that describes the attribute. When that concept fits into a scientific theory, it will be as a unique, single parameter. Campbell holds that any such measurement process will involve many concepts and so, requires laws relating many theoretical parameters. Each act of measurement must be explained by the joint functioning of many scientific laws. Thus, measurement processes cannot be adequately described by a single theoretical parameter (as required by definitional operationism).

Next, he gives an argument from “scientific experience” (Campbell 1969/1988, 33). Sometimes in scientific day-to-day practice, a researcher discards readings from her instrument that she takes to be in error. Suppose she does so when the readings suggest the instrument indicates the phenomenon is not present. She can only suspect error if she believes that phenomenon is real and present. Such reasoning would not be possible if definitional operationism were correct. On definitional operationism, the procedure completely defines the conditions of application of the concept. If those conditions are not met (e.g., the expected recordings are not observed), then the phenomenon is not present. Campbell holds that definitional operationism cannot make sense of researchers’ “criticism of method” and “improvement of method” (Campbell 1969/1988, 33).

The question of how to integrate evidence from different means of detection (or operations) becomes central. Campbell holds that researchers find themselves in a very human predicament: “we have only *other invalid* measures against which to validate our tests; we have no ‘criterion’ to check them against” (1969/1988, 33). There is no direct access to the ‘criterion’ or phenomenon (and so no infallible procedure for checking ‘criterion validity’ for our test). In recognizing our human limitations at accessing certain phenomena, as in much of psychology, we can still validate our tests with what limited resources we do have, by cobbling together imperfect, error-prone measures to arrive at joint validation. Such reasoning leads Campbell to endorse the strategy of multiple operationism. He notes: “great inferential strength is added when each theoretical parameter is exemplified in two or more ways, each model being as independent as possible of the other, as far as the theoretically irrelevant components are concerned” (1969/1988, 33).

Despite casting a wider view of science than the positivism common in the social sciences at the time, Campbell assumes a universal picture of philosophy of science characterized by laws. One might worry that it is less applicable to fields like psychology. He also emphasizes the role of the theoretical terms that feature in scientific laws in the theoretical argument, though I think his view of theories is rather piecemeal. It would fit with his later development of evolutionary epistemology, and it is consistent with his view on construct validity that builds on Cronbach and Meehl’s account. Still, it is worth noting that Wimsatt (2007, 77–78) much more radically rejects theories as axiomatic and other idealizing assumptions about science as deductive or algorithmic. Such a philosophy of science is only suitable for “LaPlacean demons” who can afford to wait for slow, cautious, and algorithmic solutions (Wimsatt 2007, 77).

Wimsatt (1981/2007, 53) takes Campbell’s view to be significant for theoretical descriptions of attributes. He interprets Campbell’s view as one in which each operation is “contingently associated with the thing measured” but is too imperfect to define it (Wimsatt 1981/2007, 53). When multiple operations are viewed in concert, however, they can “give a more accurate and complete picture” of the measurand (Wimsatt 1981/2007, 53). Crucially, Wimsatt’s commitment to realism (e.g., Wimsatt 2023; Eronen 2015) bolsters this claim. If the thing to be measured is complex and has multiple attributes, then we should not expect any single measurement process to capture all of these attributes accurately. The complete picture of the phenomenon needs to be constructed from many fallible processes. Further, Wimsatt notes that any inference from measured quantity to the existence of a robust property will involve idealization, often treating attempts to practically minimize known systematic error in the instrument readings as absent. For definitional operationism, this practice is not justified for the operation defines the quantitative property. For Wimsatt, they are strengths of robustness as a strategy: distinct measures involve different theoretical idealizations and so, error can be understood and (to some extent) controlled (1981/2007, 54).

### Construct Validation

In “Convergent and Discriminant Validation by the Multitrait-Multimethod Matrix,” Campbell and Fiske (1959) build on the concept of construct validity laid out by Cronbach and Meehl (1955). Cronbach and Meehl focused on how empirical relations between empirical variables could be used to assess theoretically predicted relationships between a web of interconnected theoretical constructs. On Campbell and Fiske’s view, a test produces data as “a union of a particular trait content with measurement procedures not specific to that content” (1959, 81). That is, data produced in measurement is caused by both some attribute and the measurement process itself. To evaluate the extent to which the test scores reflect the attribute of interest (i.e., validity), the researcher must employ strategies to disambiguate these influences.

Campbell and Fiske (1959) offer a set of empirical evaluations of test score correlations in Multitrait-Multimethod (MTMM) matrices. Social science often uses self-report methods to measure social attitudes, traits, or behaviors (e.g., the trait courage). Researchers can also use different methods to assess the same trait. One might compare an individual’s courage by asking for their own self-report and asking their friends, family, and colleagues to provide third-person reports. Exploiting this flexibility, researchers can systematically compare correlations in scores that employ the same method and/or are intended to measure the same trait. As construct validity fundamentally involves convergence from independent methods, it can be assessed by examining correlations between *different* methods intended to measure the *same* trait. When these correlations are large, there is evidence for validation of the test interpretation (Messick 1989; Truijens et al. 2019).

However, validation of a novel test requires discriminant validity too. When two tests are intended to measure different constructs, their scores should have low correlations. Tests can be invalidated when their scores have “too high correlations with other tests purporting to measure different things” (1959, 84). Campbell and Fiske suggest three empirical assessments of discriminant validity. A test interpretation is better validated when it has higher correlations for different-methods-same-trait than for different-methods-different-traits (1959, 82). Returning to our example, self-report and informant reports about an individual’s courage should better correlate than either with speed or errors on mathematics problems. The latter two are unrelated in both method and trait. Next different-methods-same-trait correlations should be higher than same-method-different-traits. In this case, both self-report and informant report of courageousness should correlate more strongly with each other than the correlation between self-report measures of friendliness and courageousness. Finally, there should be consistency in patterns of correlations for same-method-different-traits and different-methods-different-trait (1959, 83). For example, the former should have higher correlation among scores than the latter, due to shared method variance.

Throughout Campbell and Fiske (1959) take a fallibilist approach to their new method. They recognize that there will almost always be some correlation between different traits and methods, even when the tests are designed to measure different traits (1959, 84). Campbell and Fiske note: “In practice, perhaps all that can be hoped for is evidence for relative validity, that is, for common variance specific to a trait, above and beyond shared method variance”, where the latter is never expected to be zero (1959, 84). Shared method variance occurs when some aspect of the test or its context influences the scores, even independently of the influence of the trait. For example, the desire to be viewed positively by the researcher might influence participants’ self-reports on a variety of scales: courageousness, empathy, prosociality, and so on. They also emphasize that the multi-trait multi-method matrix itself is “an important practical *first step* in avoiding” errors of attributing variance caused by our methods to an attribute (1959, 101; emphasis added). Likewise, independence of our methods is important “[w]herever possible” because for independent methods, researchers have “no prior reason for believing that they share method variance” and thus, high correlations between scores of different methods for the same trait can be attributed to the influence of the trait on scores (1959, 103).

Campbell and Fiske’s MTMM method for evaluating construct validity aligns with Campbell’s understanding of robustness as supporting realist claims that are accessible to limited beings. We find evidence of this in an unsent letter authored by Campbell (but discussed with Fiske) that was written but not sent to Lee Cronbach. In the letter, Campbell emphasizes the importance of different measures (or apparatuses) and pushes Cronbach on whether he agrees. Campbell writes: “If you do not emphasize a difference in apparatus how do you distinguish construct validity from reliability?” (Campbell, n.d.-b). Psychologists typically distinguish validity from reliability, where the latter is the extent to which scores remain similar across different applications of the same test (e.g., different context). Reliability does not determine questions about validity: do the test scores reflect the attribute that we think they do? Presenting his own view, Campbell says: “do you disagree that all validational procedures…must involve a difference in apparatus? …I personally would go all the way: something knowable in only one manner has untestable validity, the meter readings are uninterpretable or are of equivocal interpretation. The triangulation typical of physical experimentation reduces that equivocality.” (Campbell, n.d.-b). In other words, robustness is crucial for assessing validity because it rules out alternative interpretations of our test scores. If an attribute were only accessible by one method, Campbell holds this restricts scientists from evaluating construct validity.^8^ Campbell continues: “Of course, and this is probably your main point, this independence of methods need not take the specific rigid form which we are suggesting” (Campbell, n.d.-b). Campbell is claiming that validity is only testable when there are multiple (partially independent) instruments because a single method alone cannot distinguish variance due to measurement error.

One of the other major themes in Campbell’s letter is that the MTMM analysis can help social scientists recognize potential systematic error. In the same letter to Cronbach (discussed above), he writes: “One of the major practical values I see in our criterion is, were it to be adopted that more test constructors would be made concretely aware of the role of apparatus variance in our measuring instruments” (Campbell, n.d.-b). He also argued that evaluating construct validity via MTMM can address certain kinds of researcher bias. Campbell notes: “I am convinced and I expect that you are also, that were rigorous efforts made to evaluate the construct validity of the Rorschach test and the [Thematic Apperception Task] in clinical practice that these would be found to have very low construct validity, certainly much lower than the practicing clinician would anticipate. Thus construct validity like other validity concepts should be a procedure increasing reality testing, a way of keeping people from kidding themselves” (Campbell, n.d.-b). While Campbell and Fiske aimed to provide strategies for researchers to practically evaluate construct validity and address potential systematic errors in their measures, they were ultimately disappointed with its uptake in practice (Fiske and Campbell 1992). And while Campbell was sensitive to the improvement of a measure, MTMM does not give specific guidance about how to improve a test or construct (Feest 2025). These issues have continued on in philosophy of science through engagement with Wimsatt’s view of robustness.

## Wimsattian Themes in Robustness Today

In this section, I outline several outstanding questions in the literature on robustness. What is (are) the function(s) of robustness? How should discordant evidence be addressed? Should philosophers aim for a unified account of robustness?

### The Function of Robustness

Both critics and proponents of robustness analysis have often focused on explicating Wimsatt’s claim about robustness distinguishing reality from illusion. By casting this claim in the context of confirmation, philosophers can formalize claims that successful robustness analysis increases confirmation in claims about the robust property and the sense of independence required among the methods or models.

Turning to the sense of independence among means of determination, Wimsatt (1994/2007, 197) interprets diversity as requiring independence in “the probability of failure of the different means of access.” Still, there a variety of ways that methods may fail to access the phenomenon in similar ways (Wimsatt 2007, 49–50). One might think that if the means of determination are unconditionally probabilistically independent of one another, then convergence on a property confirms hypotheses about its existence. This would mean that robustness is exceedingly rare. Confirmational independence would require the amount of confirmation provided by each method by independent of evidence from the other methods. Schupbach (2018) raises concerns that the amount of confirmation from experimental evidence from one method for a result is sensitive on previous findings. Some endorse the claim that partial probabilistic independence is sufficient (see Schupbach 2018 for discussion). Alternatively, one might consider that multiple means of detection should be theoretically independent, such that each relies on different background assumptions (Coko 2020b; Culp 1995). Sufficiently diverse means of detection might also be interpreted as methods developed from distinct historical traditions (Coko 2020b). This historical/genetic sense of diversity might track theoretical diversity, but it need not. There could be some shared background assumptions shared across distinct historical developments, if they were common across a fields’ wider background commitments or reasoned to from distinct theoretical perspectives. There is little agreement on which sense of independence is needed for a confirmatory function.

Wimsatt (2007) also stresses that robustness is a guide to what is real from what is illusory. Eronen (2015) provides an analysis of how robust detection could guide assessment of epistemic justification for ontological claims.^9^ The better the convergence across independent means of detection (within a scientific community at a given time), the better the justification that the property or entity exists (for that scientific community at that time) (Eronen 2015, 3967).

Yet, there are a variety of functions robustness reasoning can play beyond confirmation. Heesen, Bright, and Zucker (2019) and Parker (2024) endorse a version of robustness that can provide confirmation despite deep uncertainty about which of our methods are most reliable (Heesen et al. 2019) or can assess the reliability of an ensemble of models (Parker 2024). Basso (2017) argues that robustness can help evaluate measurement reliability, while Bringmann and Eronen (2016) argue it is important for assessing validity in measurement. It may also serve an exploratory function (Thompson 2022; Feest 2025).^10^ Wimsatt too thinks there is a role for robustness in exploratory aspects of research. He notes that “robustness is important in the discovery and description of phenomena and in analyzing the structure of scientific theories” because the identification of robust properties gives us candidate (descriptions of) entities/attributes that are more detectable and less sensitive to measurement error (Wimsatt 2007, 63). In fact, this exploratory and descriptive function is how Wimsatt interprets Campbell and Fiske’s focus on discriminant validity (i.e., whether measures intended to measure *distinct* traits in fact do so). In assessing discriminant validity, researchers evaluate their descriptions of the attribute by assessing potential measurement bias and “attempt to guarantee that the invariance across test methods and traits is not due to their insensitivity to the variables under study” (Wimsatt 2007, 63).

Similarly, commenting on Levins’ robustness analysis, Wimsatt states that the procedure gives “no guarantee of logical truth but, rather, a heuristic (fallible but effective) tool for discovering empirical truths that are relatively free of the details of the various specific models” (Wimsatt 2007, 56). Levins’ sense of robustness analysis can also help with “[t]heory generalization” in the context of discovery (Wimsatt 2007, 57). The diversity of functions on Wimsatt’s view is aligned with his larger epistemic framework. In a recent chapter, Wimsatt (2023) notes that sensitivity to different functions is central to his work because scientific practice itself is a goal-oriented procedure. It is no surprise then that robustness too is shot through with functional diversity.

### The Problem of Discordance

When one employs multiple methods, their results do not always converge. Stegenga (2009; 2012) calls this the problem of discordant evidence. Proponents of robustness reasoning, on Stegenga’s view, do not provide normative guidance to researchers about how to proceed given such discordant evidence (i.e., how to amalgamate it). As a result, “robustness is worse than useless” because it is “the source of the problem” (Stegenga 2009, 658). To resolve discordant evidence, Stegenga holds that researchers need to employ an amalgamation function to determine whether multimodal evidence is robust and to what extent. Stegenga (2012, 221) notes that minimally for an amalgamation function “each mode of evidence must be assessed on its quality, relevance, and salience, and the set of the modes of evidence together should be assessed on its concordance”. Stegenga (2012, 220–221) locates the problem at the lack of consensus on *a single* standardized amalgamation function among disputants in a scientific debate. However, it seems that transparency about the process of amalgamation functions could resolve most of Stegenga’s concerns about ambiguity in communication during scientific debates. Further, Kuorikoski and Marchionni (2016) argue that researchers often do have evidence about the reliability of their methods that informs how evidence from each should be weighted, even if they lack a single amalgamation function for all cases.

However, a more Wimsattian response is to suggest that the lack of a standard amalgamation function can be *epistemically fruitful*. While error is an inevitable feature of scientific investigation and the production of knowledge (Novick and Havstad 2026), it can also be a virtue of the process for limited epistemic beings like us. Two recent approaches to measurement in the geosciences have called attention to the fruitfulness of error and multiple amalgamation functions.

Watkins (2024) argues that the tendency not to calibrate or combine proxy measurements in paleoclimatology allows researchers to guard against the influence of unidentified error. Watkins breaks down two benefits for the practice of not intercalibrating proxy measures: maintaining independence of the measures and mitigating the spread of unknown error (Watkins 2024, 799). To the extent that one’s reasons for preferring independent methods is due to the likelihood of different systematic error, these two benefits align. In Wimsatt’s chain of reasoning analogy, the unknown error might impact one line of evidence supporting a proxy measurement value, but through keeping each line separate (or a “sterile” environment without calibration), errors will not “infect” the other lines of evidence (Wimsatt 1981, 66–67).

Similarly, Ohnesorge (2021) argues that geodesists resolved discordant values of two indicators of the Earth’s polar flattening in a way that is epistemically successful. Rather than forcing convergence by alignment or jettisoning one indicator entirely, Ohnesorge (2021, 245) argues that geodesists “produced novel data that grounded valuable theoretical hypotheses”. Based on theoretical virtues of fruitfulness and coherence, discordant values from two measures can be used to direct and improve future inquiry.

### Unifying Robustness?

Some philosophers of science have argued that robustness reasoning is not a unified strategy (Calcott 2011; Eronen 2015; Kuorikoski and Marchionni 2016). Distinctions among robustness reasoning might be warranted when the strategies apply in different contexts and have distinct “normative credentials” (Woodward 2006), or different justifications (McLoone et al. 2025), or distinct licenses for realist claims (Eronen 2015). For example, Kuorikoski and Marchionni (2016, 228) analyze triangulation (or determination robustness) as a strategy for “controlling for errors and biases of particular methods of determination” in data-to-phenomenon inferences (Kuorikoski and Marchionni 2016, 229). This benefit is achieved to the extent that convergence occurs across independently reliable methods (that do not share systematic error). They take triangulation to be distinct from robust theorems among diverse models (Kuorikoski and Marchionni 2016, 238).

Other philosophers of science have sought to provide a definitive, unified account of robustness, often focusing on its confirmatory function. Schupbach (2015; 2018), Dethier (2024), and Winsberg (2021) argue that a unified account of robustness can account for cases involving diversity among models, experiments, and other types of evidence, though there is some disagreement about the specifics of the account. For example, Dethier (2024, 2734) argues that there is no difference in kind between robustness of a claim across a variety of models or experiments because both rely on “precisely the same probabilistic features of evidence.” He argues that we ought to treat model reports as evidence. Rejecting this premise comes on pain of denying justification for many cases of model-based beliefs (Dethier 2024, 2740). If one accepts model reports as a type of evidence, Dethier explains the same rules of confirmation theory apply.

Jonah Schupbach’s recent explanatory account (2015; 2018), endorsed by Winsberg (2021), has been the most influential unified account of robustness. On this view, robustness centrally involves discriminating among competing explanations of the result. The basic idea is that a result is (most) robustly detected when each new means of detection rules out potential alternative explanations of the result.^11^

Schupbach (2018, 288) argues methods are *sufficiently* diverse when they can explanatorily discriminate among alternative hypotheses, which he explicitly defines as ‘RA-Diversity’:

Means of detecting R are RA-Diverse with respect to potential explanation (target hypothesis) H and its competitors to the extent that their detections (R1, R2, …, Rn) can be put into a sequence for which any member is explanatorily discriminating between H and some competing explanation(s) not yet ruled out by the prior members of that sequence.

RA-Diversity is met to a greater extent when any sequence of detection a result is explanatorily discriminating between the target hypothesis and live, competing hypotheses. As a result, the explanatory account of robustness reasoning in practice resembles a series of experiments (or modeling activities) to (dis)confirm hypotheses. RA-Diversity is relative to a particular hypothesis and a graded notion. Means of detection that fail to rule out an alternative explanatory hypothesis will diminish the diversity of the set. Schupbach offers a Bayesian analysis of RA-diversity, which aims to guarantee that higher RA-diversity among means of detection results in higher confirmation for the target hypothesis.

Interestingly, we find evidence of the idea that robustness reasoning can rule out alternative hypotheses in Campbell’s work more than in Wimsatt’s. In Campbell’s notes for the famous MTMM matrix paper (Campbell and Fiske 1959), he notes this potential function of triangulation. Campbell writes: “Triangulation—as binocular or monocular parallax reduce the number of competing alternative interpretations of a single retinal image” (Campbell, n.d.-a). It appeals both to matching patterns between different perspectives (methods) and the reduction in competing alternative interpretations of the data. He also writes: “Triangulation -eliminates-or reduces- competing hypotheses-” (Campbell, n.d.-a). However, in the published paper with Fiske, Campbell does not explicitly refer to this hypothesis-elimination function of robustness. I do not think this omission is because Campbell ultimately rejected the idea. It fits with his and Fiske’s larger concern about distinguishing score variation that can be attributed to the construct rather than our measures. However, there is some reason to think Campbell’s approach to hypothesis-elimination is framed differently than Schupbach’s version. To limited beings, it matters whether researchers are investigating *particular* sources of error (motivated by an alternative hypothesis) or are not even able to distinguish error in measurement from error in characterization of the phenomenon (Campbell and Fiske 1959).

Consider how these notes fit with Campbell’s idea of evolutionary epistemology in which knowledge is gained by the use of various mechanisms of knowing that operate on the basis of “blind variation” and “selective retention”, just as adaptationist views of evolutionary processes (Campbell 1974, 402). On this view, knowledge processes generally work by ruling out specific alternative hypotheses and selectively retaining those that remain. However, this is an ongoing process of validation that will often never be complete (Campbell, n.d.-a). Like Wimsatt, he emphasizes that blind trial and heuristic principles are how limited beings generate knowledge from epistemically imperfect positions (Campbell 1974, 412). He also sees continuity in other forms of knowledge processes, including the development of vision and animal imitative learning (Campbell 1974). Finally, Campbell is interested in how learners come to consider these alternative processes, not in the logical analysis of ruling out alternatives (Campbell 1974, 400–401), which fits more with Wimsatt’s approach to science as a human activity than Schupbach’s analysis of the logic behind (successful) robustness reasoning.

## Robustness Reasoning as a Heuristic

Rather than attempting to provide a unified account of robustness, I propose that we return to Wimsatt’s idea that robustness reasoning is a heuristic (e.g., 2007, 89). It is a rule of thumb that can be applied and adapted to specific problems in a given context. It is not a recipe for producing successful science (e.g., true claims about what entities exist, distinguishing competing hypotheses by producing discriminating evidence with new methods). Instead, it is one strategy among many that researchers may employ that has shown some past success for exploring one’s characterization of a phenomenon, confirming a claim, or evaluating the reliability of an ensemble of models. Further, as a heuristic strategy, robustness reasoning need not be evaluated in the absence of other relevant background knowledge or evidence that might bear on any particular assumption (Lloyd 2015; Winsberg 2021).

While Wimsatt (1981/2007, 44) does note that robustness analysis is a “family of criteria and procedures”, he does claim that all robustness analysis has a common set of features (Sect.  Robustness as a Guide for Epistemically Limited Beings). Wimsatt himself endorses a kind of unity: “One may ask whether any set of such diverse activities…is usefully combined under the umbrella term *robustness analysis*. I believe that the answer must be yes” (Wimsatt 1981/2007, 46). Wimsatt provides two reasons for a family resemblance category. He claims that all instances of robustness reasoning have the same broad epistemic function in distinguishing what is “ontologically and epistemologically trustworthy and valuable” from what is not (Wimsatt 1981/2007, 46). It is worth noting that this abstract characterization of the function might capture the different functions mentioned in Sect. The Function of Robustness. Robustness reasoning may at different times serve many specific functions (exploratory, confirmatory, ontological) that fall under this broad characterization. Second, each method makes use of some sense of independence between the methods (Wimsatt 1981/2007, 46). The failure of independence is one type of systematic error (“illusion”) that can result from robustness reasoning.

Heuristics involve rules-of-thumb, or other shortcuts, in reasoning to save cognitive resources. We use heuristics because they often work to solve our problems. Heuristics can also be applied to new problems outside their original domain to help us find a solution. To do so, heuristics must be “retuned, re-modulated, re-contextualized” for current problem context (Wimsatt 2007, 10). Even these error-prone procedures can guide us to better epistemic practices. For Wimsatt, error can also sometimes be fruitful. As he puts it, our systems can function with a “metabolism of error” in which error can be “sources of creative elaboration” (Wimsatt 2007, 22). We need not aim to fully minimize error, even as we recognize that error it is impossible to avoid (Wimsatt 2007, 11). By building a system of knowledge about our heuristics, how they work, and when they fail, we can permit error into our epistemic system. As Wimsatt says, “Maybe errors are okay—or tolerable—as long as they aren’t too frequent, and if we have good ways of remembering, detecting, and recovering from common ones; learning from patterns of our mistakes and effectively maintaining, refining, and teaching what we have learned” (Wimsatt 2007, 22). As living and social creatures, we encounter epistemic problems of similar form to those encountered before. We use heuristics to guide our problem-solving. Our inferential practices are themselves, for Wimsatt, partly the result of our own previous experiences and even learned practices adapted from previous generations (Wimsatt 2007, 22).

Here I will suggest that interpreting robustness as a heuristic addresses three major concerns in the literature: (i) whether robustness reasoning can fail, (ii) the problem of discordant evidence, and (iii) the role of alternative hypotheses in robustness.

Just as Wimsatt’s four-step schema for robustness reasoning encourages researchers to consider the conditions under which invariance breaks down, so too should we assess the limits of robustness as a heuristic. No heuristic guarantees success in a particular case. Instead, we may be likely to fail in our tasks when using heuristics in characteristic ways. Wimsatt (1981/2007, 71) recognized that there can be “illusions of robustness.” Specifically, he focuses on those cases where the means of determination are not in fact independent despite our belief that they are (Wimsatt 1981/2007, 71). However, there is another form of illusory robustness. In some cases, we may assume that our methods produce evidence about the same phenomenon, when in fact they do not (Thompson 2022). Just as Campbell and Fiske (1959) suggest, a lack of supporting evidence may sometimes require reconceptualizing the phenomenon. Explicating these illusions is an important part of the heuristics view. It also focuses more on what commonalities there are in robustness reasoning across scientific practices than treating robustness as a success term.^12^

Consider now the “problem” of discordant evidence. The heuristic view of robustness reframes Stegenga’s claim that the problem of discordant evidence challenges the “general applicability of robustness” (Stegenga 2012, 211). On Wimsatt’s view, investigating the scope of invariance of, say, an experimental result means one will *often* find discordant evidence. To identify the scope, we need to know under what conditions the invariance *fails* to hold. As epistemically limited beings, identifying the conditions under which invariance does not hold is important for identifying where our heuristic breaks down. Instead of holding that robustness strategy has a problem that prevents its usage, researchers should use this breakdown of the heuristic to seek more knowledge (Novick and Havstad 2026), namely, to explain why these changes in conditions produce variance in the result.^13^ Still, Stegenga’s challenges may be correct to point out that robustness reasoning *alone* cannot guide scientists in assessing the discordant evidence. But on the heuristic view of robustness reasoning, we would not expect it to do so. For example, Kuorikoski and Marchionni (2016, 239) hold that researchers may often have information about the reliability of each method and how to weight evidence from each, even if a single amalgamation function for all contexts is absent. Researchers can and should employ other information and heuristics when robustness as a strategy breaks down.

Another issue the heuristic view helps address is the different roles of alternative hypotheses in Wimsatt’s and Schupbach’s accounts. Wimsatt’s four-step schema for robustness reasoning does not rely on the researchers already specifying alternative hypotheses. The active verbs indicate that researchers should first be “searching” and “identifying” prior to explaining. Instead, researchers vary features of their means of detection without regard for distinguishing particular hypotheses. Information that changing this value of a variable or altering this background condition in an experiment does not impact the result (the invariance holds) is just as useful as information that it does (the invariance does not hold). The process may generate alternative hypotheses through identifying the scope of the invariance and searching for features or conditions that explain it.

On the other hand, Schupbach’s explanatory account of diversity in robustness is always evaluated relative to a set of competing hypotheses. Greater diversity among the means of detection and thus greater confirmation to an explanation when the researcher varies the means of detection economically—the minimal needed means of detection in order to rule out plausible alternative explanations. It is not strictly required that the researcher already conceptualize the alternative explanations in advance (one could always get lucky with a diverse sequence), but it would be beneficial to do so. From Schupbach’s view of robustness, we get a picture of researchers carefully determining which alternative explanation to investigate and then identifying the best method to produce evidence discriminating between the two explanations before conducting any analysis or experiment. Robustness reasoning ends up procedurally as a sequence of experimental hypothesis-testing, simply applied more broadly to other means of detection.

Do scientists always set up experiments (or other means of detection) to discriminate among competing hypotheses? Should they? If answered affirmatively, Schupbach’s view of robustness provides a compelling account of the benefits of this practice. However, I do not think that robustness reasoning in science always works this way. For those convinced that scientists also do exploratory work (Feest 2025; Steinle 1997), there is room for philosophy of science in practice accounts of cases where researchers have salient alternative explanations in mind and those in which they do not.

While Schupbach is one of the more developed unified accounts of robustness analysis, it is common among this approach to adopt a form of argument. Any proposed unified account must explain all cases of robustness analysis accepted in the philosophical literature. Any failure to apply the account to a given case must be explained as the case not really being an instance of robustness in the first place (see also Hudson 1999). Debates between proponents of different unified accounts focus on which account gives the best interpretation of the paradigmatic cases. For example, Schupbach claims that the explanatory account of robustness reasoning “is truer to standard scientific cases of [robustness analysis]” (Schupbach 2018, 289).

Still, Schupbach’s arguments rely too much on their ability to explain a particular sequence of Brownian motion experiments. For example, he argues varying the use of pollen granules from inorganic materials in experimentation helped rule out alternative explanations of the observed particle movement and supports the hypothesis of Brownian motion. However, Coko (2020a) provides an alternative understanding of robustness reasoning in Perrin’s theory of Brownian motion that proceeded first qualitatively and then quantitatively. A failure to quantitatively triangulate would also not be disconfirming of the hypothesis nor confirming of the alternative, according to Coko’s (2020a) interpretation of Perrin. Rather, it could be attributed to a variety of auxiliary assumptions. Further, even if Schupbach’s account of Perrin’s reasoning is correct, it need not *in fact* be paradigmatic of all robustness reasoning.

Other cases of robustness reasoning do not rely on the explanatory notion of diversity. As Heesen, Bright, and Zucker (2019) argue, methodological triangulation in W.E.B. Du Bois’ sense involves using multiple methods to investigate the same question in conditions of uncertainty about the reliability of each individual method. Heesen, Bright, and Zucker argue that the strength of methodological triangulation (among methods of uncertain reliability) will outperform the use of an individual method (even a more reliable one). To do so, Heesen et al. appeal to Condorcet’s Jury Theorem. It involves giving each method a “vote” on some result and taking the answer provided by the majority to be the result triangulated upon. Parker (2024, 44–49) has also recently argued that robustness reasoning in climate science among model ensembles takes this form. Parker (2024, 46) argues that convergence or divergence in outcomes or predictions can provide evidence about whether the set of models are highly or moderately reliable, which does not involve ruling out any alternative hypotheses that explains the outcome or result.

In another case, O’Loughlin (2021) applies Lloyd’s account of model robustness. Briefly, Lloyd’s (2015) view is that climate ensemble models share some core (causal) assumptions while other auxiliary assumptions differ between each model. Model robustness occurs when an ensemble of models with a common core make a similar prediction or retrodiction and when auxiliary assumptions are supported by observational evidence, the core assumptions are supported by experimental evidence, and the observations of the predicted variable confirm the predicted values. Here the causal explanation of an outcome, not merely its prediction, is given empirical support.^14^ O’Loughlin argued that Lloyd’s account of model robustness better analyzes the practice of some climate scientists using climate ensemble models to identify relevant causal processes. O’Loughlin and Parker independently offer objections to Schupbach’s argument: provide a better interpretation of this case according to the favored unified account or explain why this is not *really* a case of robustness after all. However, the heuristic view of robustness offers an alternative—one that I think O’Laughlin and Parker both adopt—there are different functions of robustness reasoning in different contexts.

Similarly, Hudson’s (2014) critiques of robustness adopt the unified view as a constraint that shapes the discourse. Hudson analyzes what are taken to be paradigmatic cases of robustness reasoning and argues that they are not in fact about robustness. Rather, these cases can be explained as researchers searching for sufficiently reliable methods, not confirming claims about the existence of a property or entity. However, re-interpreting some paradigmatic cases is not sufficient to show robustness reasoning is descriptively inadequate in its account of scientific practice nor that it fails to achieve other aims. This argumentative strategy relies on a unified account of robustness, which many are increasingly rejecting (Calcott 2011; Eronen 2015; Woodward 2006).

There are also potential benefits to re-thinking Schupbach’s account in a heuristic framework. It is not a complete picture of robustness to show how the Bayesian interpretation guarantees confirmation when RA-diversity is met. Instead, a good account of the explanatory robustness heuristic needs to say much more about contexts in which the strategy works and when it breaks down. Plausibly, these might include that the strategy does not require (complete) independence of theoretical assumptions, does require a specific alternative hypothesis to be tested, and does require that the series of tests/analyses happen in sequence. From a Wimsattian perspective, we might also ask: how do researchers arrive at these alternative hypotheses? How do they determine which series to investigations to run and in which order? What do researchers do when they are not able to rule out some of the alternative hypotheses? What if they believe that their experimental manipulation was imprecise? On the latter question, Feest’s (2025) analysis of Garner, Hake, and Erikson’s (1956) converging operations is instructive.

## Conclusion

I have suggested that we should give up on the project of identifying a unified account of robustness that secures only its confirmatory function and applies to all cases in which researchers use robustness as a strategy. That also means giving up on the potential strengths of a unified account; robustness should not be treated as “an epistemic virtue that ensures objectivity” (Stegenga 2009, 651). We can give up the project of a unified account of robustness reasoning by returning to the heart of Wimsatt’s (and Campbell’s) insights. Human researchers in a messy, complex world with imperfect tools will need heuristics to guide their empirical investigation and understanding. Robustness reasoning is one family of heuristic strategies that are loosely held together. Robustness reasoning may help researchers in developing a fuller characterization of the object of inquiry, judge the likelihood of influence from a measure on data production, or sort reliable, secure claims from those that are less so. Different problems and contexts will allow different applications of robustness heuristics. The benefit of moving to a Wimsattian frame of robustness as a heuristic would be to trace how the heuristic changes in different contexts, identify the conditions under which each fails, and seek other strategies for those cases.

## Data Availability

No datasets were generated or analysed during the current study.

## Footnotes

1 Personal communication with William Wimsatt (on July 28, 2026).

2 Thanks to a reviewer for this reference.

3 Now, Wimsatt would emphasize that these failures can be explained through cross-checking and analyzing the differences among the methods (personal communication; July 28 2026).

4 Here I adopt the language from Wimsatt (1981) and Soler (2012): means of determination is meant to broadly encompass derivations, models, methods, and measures.

5 One reading of Bridgman’s operationism is that a given concept is completely defined by its operational definition. As such, investigations employing different means of detection (different operations) will produce information about different concepts. While this may not be Bridgman’s considered view (Feest 2025), it is how Donald Campbell interpreted the logical positivists (Campbell 1988a, b).

6 Stegenga (2012) calls this ‘discordant evidence’.

7 Campbell holds that operational definitions are “abbreviated descriptions used for designation” that researchers use for “entities given real status, and hence with innumerable attributes, known and unknown” (Campbell 1996, 32). Campbell never argues for realism and in fact, sometimes he simply assumes it (Campbell 1977). Wimsatt, too, has continuously affirmed his realist perspective (i.e., Wimsatt 1981, 2023). Novick and Havstad (2026) argue that Wimsatt can be interpreted as adopting a realist *attitude* in Arthur Fine’s sense.

8 Campbell’s note has some interesting connections to current debates about construct validity. It is possible that Campbell holds the measure is valid, but it is not something we could test or know. Alternatively, one might think that untestable validity is not validity at all. If validity is not a property of tests but rather of score interpretations or arguments (e.g., Messick 1989), then this kind of inaccessible fact has no relevance to validity claims. It cannot be assessed until another method purports to access the same phenomenon.

9 See Kuorikoski et al. (2010, 2012) and Lloyd (2015) for an explication of robust theorems and realist claims about causal processes.

10 Here the exploratory function need not be observational. Much experimental work in psychology involves first attempting to adequately describe and characterize the attribute or phenomenon of interest.

11 For those who object to Schupbach’s use of ‘detection’ here, it could be replaced with ‘determination’ following (Soler 2012).

12 Soler (2012) also situates Wimsatt’s view of robustness within the philosophy of science in practice movement.

13 Similarly, Kuorikoski and Marchionni (2016, 239) respond to Stegenga’s problem by noting that in cases where justification cannot be given for weighing evidence from different methods, it would be rational to suspect judgement rather than rejecting the hypothesis. Discordant evidence is a sign that more inquiry is needed.

14 Similar arguments about robust theorems are made by Kuorikoski et al. (2010).
